# Sensitivity Limits for *in vivo* ELISA Measurements of Molecular Biomarker Concentrations

**DOI:** 10.18416/ijmpi.2017.1706003

**Published:** 2017-06-22

**Authors:** John B. Weaver, Yinpeng Shi, Dylan B. Ness, Hafsa Khurshid, Anna Cristina S. Samia

**Affiliations:** a Department of Radiology, Dartmouth-Hitchcock Medical Center and the Geisel School of Medicine, Dartmouth College, Hanover, NH, USA; b Department of Physics, Dartmouth College, Hanover, NH, USA; c Thayer School of Engineering, Dartmouth College, Hanover, NH, USA; d Department of Chemistry, Case Western Reserve University, Cleveland, OH, USA

## Abstract

The extremely high sensitivity that has been suggested for magnetic particle imaging has its roots in the unique signal produced by the nanoparticles at the frequencies of the harmonics of the drive field. That sensitivity should be translatable to other methods that utilize magnetic nanoparticle probes, specifically towards magnetic nanoparticle spectroscopy that is used to measure molecular biomarker concentrations for an “*in vivo* ELISA” assay approach. In this paper, we translate the predicted sensitivity of magnetic particle imaging into a projected sensitivity limit for *in vivo* ELISA. The simplifying assumptions adopted are: 1) the limiting noise in the detection system is equivalent to the minimum detectable mass of nanoparticles; 2) the nanoparticle’s signal arising from Brownian relaxation is completely eliminated by the molecular binding event, which can be accomplished by binding the nanoparticle to something so massive that it can no longer physically rotate and is large enough that Neel relaxation is minimal. Given these assumptions, the equation for the minimum concentration of molecular biomarker we should be able to detect is obtained and the *in vivo* sensitivity is estimated to be in the attomolar to zeptomolar range. Spectrometer design and nonspecific binding are the technical limitations that need to be overcome to achieve the theoretical limit presented.

## I. Introduction

Molecular biomarkers are being sought for many diseases and conditions. The promise of diagnosing and characterizing physiology and pathology using a blood or saliva test is so powerful that enormous resources are being devoted toward that goal. The latest blood tests are sufficiently sensitive [1] but a vast majority of potential biomarkers lack sufficient specificity because very few molecular markers are unique to a particular disease state. Most biomarkers, even those that are strongly associated with a specific disease, are produced by so many other sources that increased concentrations in blood are often not indicative of the presence of disease or of disease progression. Many biomarkers are associated with cancer. Many are critical to the growth of malignancies: immune cytokines (interferons and interleukins), angiogenesis markers, like vascular endothelial growth factor, VEGF, and extracellular matrix, ECM, remodeling enzymes such as matrix metalloproteinases, MMPs, and lysyl oxidase, LOX. All of those factors are also present in normal tissues and increases can result from minor conditions like a cold or a cut so their increase in blood tests have been notoriously poor prognosticating factors. On the other hand, local disregulation is highly specific especially for effector molecules that perform specific tasks necessary to the pathological progression. For example, local up-regulation of VEGF is critical to cancer [2, 3], but it is also up-regulated in many other conditions, such as wound healing [4–6] and even in trauma as minor as puncture with a needle stick used to obtain a blood sample [5, 6]. Clearance is also variable [7] so serum VEGF has little diagnostic impact [3, 8] but local VEGF up-regulation is far more specific [2, 3]. The local concentration of biomarker is also much higher than those in serum [7] where it has been diluted and attenuated by clearance.

The other approach for measuring *in vivo* biomarkers is biopsy, which provides the local concentration. Although, tissue heterogeneity limits the influence of biopsy results [9] it will undoubtedly remain the basis of many diagnoses and will probably be utilized more because it provides a detailed genetic profile [9]. However, repeated biopsy is problematic at best so monitoring biomarker concentrations is not feasible using biopsy.

*In vivo* measurements of cell surface receptor numbers have been reported using positron emission tomography, PET, [10] and optical methods [11, 12] with kinetic modeling. But kinetic modeling methods are unable to measure concentrations of free molecule biomarkers like cytokines, chemokines, or hormones. In addition, PET is limited for longitudinal measurements, is expensive and inflicts a radiation dose. Optical methods [11–15] are limited to shallow depths.

Further, technology using magnetic resonance imaging, MRI, instead of magnetic nanoparticle spectroscopy to detect binding has measured blood levels of cardiac biomarkers longitudinally over 72 hours [16]. However, centimeter size probes were necessary and MRI is expensive and not readily amenable to point of care applications.

**Figure 1:**
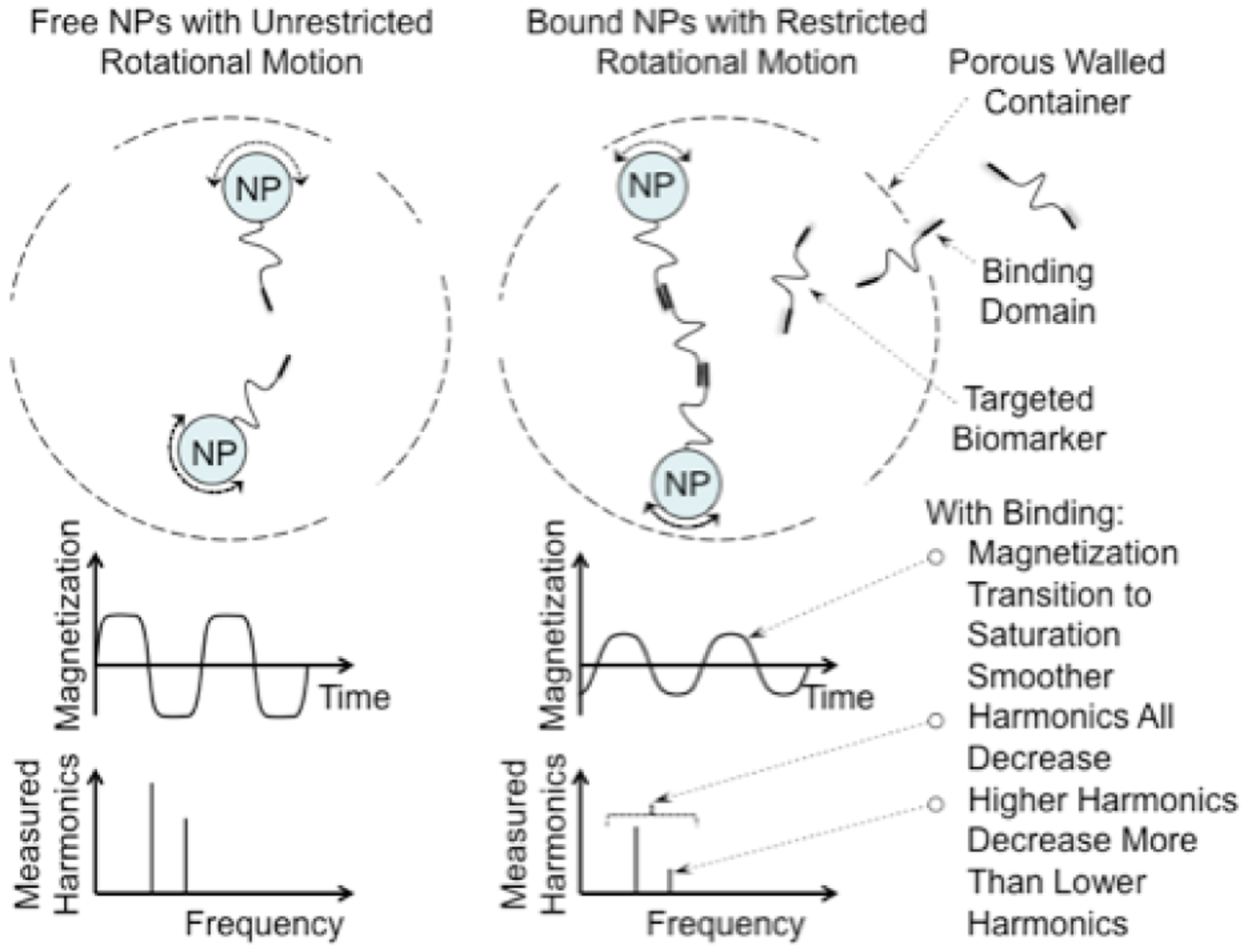
The physics of the MSB measurement. When the NPs are not bound they rotate freely and align themselves with the applied field quickly resulting in sharp corners as the magnetization saturates. The sharp corners produce large signals at the harmonics. The biomarker binds the NPs together reducing their rotational freedom, reducing the speed the NPs align with the applied field rounding the corners and reducing the harmonics and the ratio of the harmonics.

Magnetic spectroscopy of nanoparticle Brownian motion, MSB, is a magnetic nanoparticle, NP, based method that has been adapted to mimic the ubiquitous enzyme-linked immunosorbent assay, ELISA [17], method of quantifying molecular concentrations in solutions and *ex vivo* samples. Because it forms sandwiches of reporting molecules surrounding the biomarker as in ELISA the MSB based method has been termed “*in vivo* ELISA” because it can be used in vivo which the traditional ELISA method cannot. The *in vivo* ELISA method uses NPs coated with aptamers or affibodies that bind two independent epitopes on the biomarker molecule so the NPs form a sandwich around the biomarker molecule thereby restricting the rotational motion of the NPs [18]. The restricted motion translates to changes in the observed MSB signal (Fig. 1) [19].

## II. Methods and Results

The maximum sensitivity of methods like *in vivo* ELISA can be approximated using the noise levels of the MSB or MPI measurements, estimates of the signal change produced by binding and the chemical binding constants. The analysis is presented below.

Specifically, the maximum sensitivity for *in vivo* ELISA can be estimated using three assumptions: 1) the signal difference produced by binding should be larger than the intrinsic noise in the system; 2) the equilibrium constant for binding governs the proportion of bound NPs for a given concentration of molecular biomarkers; and 3) the total number of NPs must be conserved.

The minimum number of detectable NPs, *N*_min_, is essentially the noise limit of the experiment [20]. The minimum concentration measurable using *in vivo* ELISA is obtained when the change in signal is at the noise limit. Therefore, the product of the number bound, *N*_bound_, and the change in measured signal between the unbound and bound NPs, Δ*S*,

(1) $$N_{\text{min}}=N_{\text{bound}}\cdot \Delta S$$

where *N*_min_ is the minimum detectable number of NPs, *N*_bound_ is the number of bound NPs and Δ*S* is the proportional change in signal from a NP that occurs with binding, 0 *< S <* 1. The coil geometry and coupling factors are subsumed in the minimum number of NPs detectable. Although the limit for current spectrometers is on the order of 100 nanograms of iron [21], the ultimate limit is much lower. The minimum detectable number of NPs has been estimated in several publications and is essentially 1 pgram of iron for a 10 min measurement of NPs in a liter volume [20].

The proportion of NPs bound is determined by the concentration of molecular biomarker, [*B*], the concentrations of the bound and unbound NPs, [*N*_bound_] and [*N*_unbound_] respectively, and the disassociation constant for binding, *K*_*d*_ :

(2) $$K_{d}=\frac{\left[N_{\text{unbound}}\right][B]}{\left[N_{\text{bound}}\right]}.$$

The total of the bound and unbound NPs remains constant:

(3) $$N=N_{\text{unbound}}+N_{\text{bound}}.$$

These three relationships can be reduced to an equation that can be used to estimate the minimum detectable concentration of the biomarker:

(4) $$[B]=\frac{K_{d}N_{\text{min}}}{N\Delta S-N_{\text{min}}}.$$

A slightly simpler relationship is obtained by assuming the number of NPs in the probes used is much larger than the noise, *N* Δ *S* ≫ *N*_min_:

(5) $$B]≃\frac{K_{d}N_{\text{min}}}{N\Delta S}.$$

Or if the size of the NPs used to measure the minimum detectable number is the same as the NPs used to measure the biomarker concentration then the weight per NP cancels leaving the ratio of the weights of NPs *W*_min_ and *W* respectively:

(6) $$[B]≃\frac{K_{d}W_{\text{min}}}{W\Delta S}.$$

Several observations should be made: First, the smaller the intrinsic noise in the system, *N*_min_, the higher the sensitivity as expected. Similarly, higher binding affinity (smaller *K*_*d*_) yields more sensitive detection. At first it appears that the inverse relationship with the total number of NPs, *N*, is anomalous because the percentage change n signal for a given concentration, [*B*], increases for smaller *N*. However, NP binding is assumed to not impact biomarker concentration because the probe volume is relatively small so, [*B*], is constant over the probe volume no matter how many NPs are present. Therefore, the absolute magnitude of the change in signal increases with increasing *N* as in Eq. (4) and Eq. (5). However, *N* is limited by the probe volume and the dynamic range of the detection system: if the probes are limited to ~ 100 *μ*gram of nanoparticles, *N* is ~ 10^12^. In the original paper, the NP binding that was shown to be able to provide molecular concentration used 150 *μ*gram samples [18]. If the dynamic range of the system is 16 bits, *N*/*N*_min_ is limited to 6.6 · 10^4^ after which thermal noise reflected in *N*_min_ ceases to limit the sensitivity and quantization noise dominates. A dynamic range of 24 bits allows *N*/*N*_min_ to increase to 10^7^ and 32 bits allows *N*/*N*_min_ to increase to 4 · 10^9^ before the thermal noise is eclipsed by truncation or quantization noise. Eq. (4) and Eq. (5) are predicated on thermal noise limiting the measurement. If *N*/*N*_min_ exceeds the dynamic range of the system then *N*/*N*_min_ should be replaced by the effective dynamic range to estimate the minimum detectable concentration.

One can estimate the limiting observable concentration using the limiting sensitivity of MPI measurements. We use Eq. (5) and Eq. (6) and assume *N*/*N*_min_ is limited by to a 32 bit dynamic range, so *N*/*N*_min_ values of 10^8^ to 10^9^ are possible before quantization errors begin to dominate. Thirty two bits is probably near the limit of what can be achieved in ADC dynamic range just as the 1 pgram is the limit of what can be detected. Typically, antibody *K*_*d*_ values are in the 10^−11^ molar range for antibody or affibody binding and Δ*S* is 1 when the NP is bound to something of much greater mass, e.g., the probe shell, and the NP is large enough that Néel relaxation is minimal so the NP’s signal essentially disappears with binding. So Eq. 5 suggests the minimum concentration of molecular biomarkers obtainable with reasonable assumptions is on the order of 10^−19^ M (0.1 aM or 100 zM); 100 zM is ten thousand molecules per liter. The assumptions for that estimate include minimal electronic noise in the equipment, small probe volume, a liter size tissue volume, 32 bit dynamic range, and roughly 100 *μ*gram of NPs.

As the spectrometers improve and the sensitivity to iron approaches the sensitivity limit, the sensitivity to molecular biomarkers will approach the 10^−19^ M, 100 zM, limit. The other factors that could limit the sensitivity must be addressed as the sensitivity is improved: e.g., stability of the nanoparticles to aggregation and temperature variation. But the in vivo sensitivity limit of 100 zM is remarkable by any standard.

Hormones are in the 10^−9^ M concentration range [22]; cytokines regulating immune response are in the 10^−12^ M range [22] so both are well within the capability of *in vivo* ELISA through an MSB approach. Specific DNA and RNA fragments from pathologic cells might be within reach of this technology but polymerase chain reaction, PCR, is able to duplicate DNA sequences enabling very small concentrations to be detected even without methods of measuring those concentrations directly. Sensitivity sufficient to measure aM to zM concentration suggests entirely new and different classes of molecular biomarker might be monitored using this technology.

## III. Conclussions

*In vivo* ELISA based on MSB spectroscopic methods is a technique of measuring the concentrations of free molecular biomarkers *in vivo* using microscopic probes made of magnetic NPs targeted to bind the biomarker inside hollow, porous shells. The rotational freedom of the magnetic NPs changes as they are bound together by the biomarker producing a signal change measurable using magnetic nanoparticle spectroscopy tuned to Brownian motion termed MSB. The sensitivity of reversible *in vivo* ELISA was estimated to be sufficient to measure 100 zM concentrations of molecular biomarkers. This level of sensitivity would allow hormones, cytokines and enzymes to be measured using reversible binding. Spectrometer design and nonspecific binding, i.e., aggregation, are currently the technologies that limit the sensitivity and that need to be overcome to achieve the theoretical limit presented.
